# p53-dependent apoptosis is essential for the antitumor effect of paclitaxel response to DNA damage in papillary thyroid carcinoma

**DOI:** 10.7150/ijms.61944

**Published:** 2021-07-11

**Authors:** Wenshuang Wu, Tao Wei, ZhiHui Li, Jingqiang Zhu

**Affiliations:** 1 Department of Thyroid Surgery, West China Hospital, Sichuan University, Chengdu, China; 2 Laboratory of Thyroid and Parathyroid Disease, Frontiers Science Center for Disease-related Molecular Network, West China Hospital, Sichuan University, Chengdu, China

**Keywords:** PTC, PTX, p53, cell cycle arrest, apoptosis

## Abstract

A functional p53 protein plays an important role in killing tumor cells. Previous studies showed that chemotherapeutic drug, paclitaxel (PTX), showed anti-tumor activity through inducing G2/M arrest and apoptosis by targeting microtubules in tumor cells. However, PTX was not sensitive to p53-inactivated papillary thyroid carcinoma (PTC) cells by inducing G2/M arrest only. Recombinant adenovirus-p53 (rAd-p53) was used to increase the level of p53, which significantly increased the sensitivity of PTC cells to PTX by inducing S arrest, G2/M arrest and apoptosis. To discuss the anti-tumor mechanism of rAd-p53 + PTX and found p53 activation was necessary for anti-tumor effect of PTX in PTC cells. There was high level of p53 in rAd-p53-treated PTC cells. rAd-p53 + PTX increased the level of p21, p-ATM and γ-H2AX and decreased the level of Cyclin D1/E1, suggesting p53 activated p21 which negatively regulated cyclins to induce S arrest response to DNA damage in PTC cells. rAd-p53 + PTX increased the levels of cleaved-PARP-1, cleaved -Caspase 3, and BAX and decreased the level of BCL-XL, suggesting p53 regulates the expression of BAX/BCL-XL to mediate DNA damage-induced apoptosis in PTC cells. Furthermore, rAd-p53 + PTX showed significant tumor inhibition in TPC-1 xenograft model, with an inhibitory rate of 79.39%. TUNEL assay showed rAd-p53 + PTX induced notable apoptosis in tumor tissues. rAd-p53 showed good sensitization of PTX *in vitro* and *in vivo* through inducing DNA damage induced-apoptosis indicated p53-dependent apoptosis was essential for the antitumor effect of PTX in PTC.

## Introduction

Papillary thyroid carcinoma (PTC) represents ~85% of all thyroid cancer cases 1. In clinical treatment, the majority of patients with localized, well-differentiated PTC patients have a good prognosis under the traditional treatment approaches, including surgery followed by thyroid-stimulating hormone suppressive therapy with/without radioactive iodine therapy, with a 10-year survival rate of >90% 2. However, some cases challenge the traditional therapy approaches. For example, patients with distant metastatic diseases that are not suitable for surgery, of which 50% of them lose the ability to take up radiation, hinder the prognosis significantly, demonstrating a 10-year survival rate of 42% 3. The radioiodine refractory disease has a more dismal prognosis, with a 10-year survival rate of 10% 4. Therefore, further basic research that focuses on pathology and pharmacology may be required for these PTC cases.

The *TP53* gene (*P53*), which encodes the p53 protein, is one of the most common and important tumor suppressor genes that inhibits the growth and development of tumors 5. Tumor cells always have instable genomic, which is associated with the accumulation of DNA damage. The main function of p53 is to repair damaged-DNA in order to prevent altered DNA being passed on to daughter cells. p53 is a transcriptional factor containing DNA binding and transcription activation domains 6. Once DNA damage in cells occurs, DNA damage results in phosphorylation of the ATM protein. Activated-ATM phosphorylates Chk2, which activates the p53 pathway to negatively regulate cell cycle proteins 7,8. p21, as the main targeted gene of p53 during cell cycle arrest, negatively regulates Cyclins to induce cell cycle arrest 9. If DNA damage is too extensive to be repaired, p53 regulates cells to undergo programmed death, including apoptosis, senescence or necrosis 6. In the DNA damage-induced apoptosis pathway, p53 initiates the expression of apoptosis-related genes, including BAX (apoptotic gene), BCL-2 and BCL-XL (anti-apoptotic genes), to regulate cell apoptosis 10,11. Once the ratio of apoptotic gene/anti-apoptotic genes increases, it causes a change in the mitochondrial membrane permeability and leads to the release of cytochrome c from the mitochondrial to the cytoplasm 12-14. Then, cytosolic cytochrome c activates Caspase 9, which activates Caspase 3 to induce apoptosis 14.

However, tumor cells are very smart. They have developed some ways to avoid the protection of p53. There are main two ways including mutating *p53* gene and inhibiting p53's function by its antagonist to inactivate p53 in tumor cells. Studies have reported that p53 is one of the most frequently mutated genes in human cancer, and p53 mutations occurs in ~50% of tumor cells 15. In the absence of p53 mutation in tumors, the function of wild type (wt)-p53 protein is frequently inhibited by its negative regulators, such as E3 ubiquitin-protein ligase Mdm2 (MDM2) and protein Mdm4 (MDMX) 16. MDM2 directly binds to p53 and inhibits its transcriptional activity, causes ubiquitination and proteasomal degradation of p53, and exports p53 out of the nucleus 16,17. MDMX is a homolog of MDM2, which is encoded by the *MDM4* gene. MDMX also directly binds to the transactivation domain of p53 and inhibits its activity, but does not cause the degradation of p53 16,18. Inactivation of p53 contributes to some functions that are helpful for tumor cell growth, including resistance to apoptosis and chemotherapy or losing the ability to repair damaged DNA to induce genomic instability 19.

In the present study, the effect of chemotherapy on the treatment of PTC was investigated *in vitro* and it was found that PTC cells were not sensitive to the chemotherapeutic drug, paclitaxel (PTX). However, using recombinant adenovirus-p53 (rAd-p53), a recombinant replication-incompetent human serotype 5 adenovirus, in which the E1 region is replaced by a human wild type-p53 expression 20, could significantly increase the sensitivity of PTC cells to PTX. Thus, the present study illustrated the molecular mechanism of antitumor action of rAd-p53 combined with PTX in PTC cells.

## Materials and methods

### Materials

PTX, dimethyl sulfoxide (DMSO), XTT, propidium iodide (PI), Hoechst 33258, and the anti-β-actin antibody were purchased from Sigma-Aldrich (Merck KGaA); anti-p53, and anti-cleaved Caspase-3 antibodies were obtained from Abcam; anti-CDK4, anti-CDK6, anti-Cyclin D1, anti-Cyclin E1, anti-serine-protein kinase ATM (ATM), anti-Chk2, anti-phosphorylated Chk2 (p-Chk2), anti-phosphorylated H2A histone family member X (γ-H2AX), anti-H2AX, anti-cleaved poly(ADP-ribose)polymerase 1 (PARP-1), anti-cytochrome C, anti-BAX, anti-BCL-2 and anti-BCL-XL were obtained from Cell Signaling Technology, Inc.; and the adenoviral vector [recombinant human p53 adenovirus injection, rAd-p53 (Gendicine™)] was purchased from Shenzhen Sibiono GeneTech Co., Ltd.

### Cell culture

The human PTC cell lines, TPC-1 and BCPAP, were obtained from The Cell Bank of Type Culture Collection of The Chinese Academy of Sciences. TPC-1 and BCPAP cells were cultured in RPMI 1640 medium supplemented with 10% fetal bovine serum (FBS) and 50 U/ml penicillin and streptomycin. Human hepatocellular carcinoma cells (HepG2) were obtained from the American Type Culture Collection and cultured in DMEM supplemented with 10% FBS and 50 U/ml penicillin and streptomycin. The cells all had authenticated using STR profiling.

### Cell proliferation assay

Cancer cells (1 × 10^4^) were cultured in 96-well plates overnight, and then treated with different concentrations of PTX (0.625, 1.25, 2.5, 5, 10, and 20 μM) and/or rAd-p53 [10^10^ viral particles (VP)] for 48 h. XTT solution (20 μl of 3.3 mM) was added into each well and incubated at 37°C for 1-2 h. The absorbance was determined in each well at 450 nm with the SpectraMax M5 Microtiter Plate Luminometer (Molecular Devices, LLC). The proliferation of the treated cells was compared with that of the control cells, which were arbitrarily assigned 100% viability.

### Flow cytometry

After treatment with different concentrations of PTX, the PTC and HepG2 cells were fixed in 70% ethanol for ≥24 h and stained with PI for 30 min. Then, after washing with 1X PBS, the stained cells were analyzed using a CytoFlex flow cytometer (Beckman Coulter, Inc.) to determine the cell cycle distribution. To determine apoptosis, control or PTX-treated cells were stained with Annexin V-FITC (green) and PI (red) for 30 min and analyzed with the flow cytometer. The percentage of apoptotic cells (Annexin V^+^/PI^-^) was determined using flow cytometry.

### Western blotting

Cells from various treatment groups were lysed in lysis buffer (Beyotime Institute of Biotechnology) with 1 mM phenylmethylsulfonyl fluoride on ice for 30 min. Then, the cell lysates were centrifuged at 12,000 x g for 15 min and the protein lysate was quantified using the Bio-Rad DC™ Protein assay (Bio-Rad Laboratories, Inc.). An equal amount of proteins (30 μg) was separated via SDS-PAGE on 6%-12% gels at 80 mV for 2 h. The separated proteins were transferred onto a polyvinylidene difluoride membrane at 100 mV for 1 h. The blots were then blocked with 5% milk and incubated with primary antibodies overnight at 4^o^C. Next, following washing with TBS with Tween-20, the blots were incubated with secondary antibodies for 1 h at room temperature. The blots were developed with enhanced chemiluminescence (EMD Millipore) and the protein bands were imaged and semi-quantified with Image Lab Software (Bio-Rad Laboratories, Inc.).

### Immunofluorescence

Cells were cultured on poly-L-lysine-coated glass coverslips and treated with different drugs for 24 h. The cells were fixed with 4% formaldehyde for 10 min, followed by incubation with the blocking buffer (1X PBS containing 3% bovine serum albumin, 2% goat serum, and 0.2% Triton X-100) for 1 h. Then, the fixed cells were incubated overnight with a primary antibody against γ-H2AX (1:100; cat. no. 9718; Cell Signaling Technology, Inc.) at 4^o^C. Next, they were incubated with Alexa Fluor-conjugated secondary antibody (Alexa Fluor® 568; 1:800; Invitrogen; Thermo Fisher Scientific, Inc.) for 1 h at room temperature. Then, the cells were stained with the nuclear dye, Hoechst 33258. Images were obtained using an inverted fluorescence microscope.

### *In vivo* PTC tumor xenograft

TPC-1 cells (5x10^6^ in 100 μl saline) were subcutaneously injected into the right flanks of 6-week-old female BALB/cA-*nu*/*nu* mice (Beijing HFK Bioscience Co., Ltd.). When the tumor size reached 100 mm^3^, the mice were randomly divided into the following groups (n = 6/group): i) Vehicle control group, mice were intratumorally injected with 100 μl adenovirus vector/100 mm^3^ every 3 days; ii) rAd-p53 group, mice were intratumorally injected with 10^10^ VP rAd-p53/100 mm^3^ every 3 days; iii) PTX group, mice were intraperitoneally injected with 30 mg/kg PTX in 200 μl saline every 5 days; and iv) rAd-p53 + PTX group, mice were intratumorally injected with 10^10^ VP rAd-p53/100 mm^3^ every 3 days and intraperitoneally injected with 30 mg/kg PTX in 200 μl saline every 5 days. Tumor volume was measured every 3 days with a caliper as π/6 x length x width x width (mm^3^). The experiment was carried out over 30 days.

### Histopathological analysis

Tumor tissues were fixed in 4% neutral-buffered formalin for 24 h and embedded in paraffin. Then, 3-5-μm thick sections were cut on a microtome. A TUNEL assay was performed with the *In Situ* Cell Death Detection kit (Roche Applied Science), according to the manufacturer's instructions, to determine the effect of different treatments on tumor cell apoptosis. Immunohistochemistry was performed by incubating the tumor sections with mouse anti-p53 primary antibody (1:50; cat. no. ab1101; Abcam), followed by HRP-conjugated secondary antibodies. Then, the slides were developed with DAB.

### Statistical analysis

Data are expressed as the mean ± standard error of the mean (SEM) and were analyzed by using the Student's *t*-test. P<0.05 was considered to indicate a statistically significant difference.

## Results

### PTC cells were resistant to PTX

Chemotherapy is a systemic therapy and is helpful for refractory solid tumors, and thus is a possible treatment for refractory PTC, including metastatic disease and radioiodine refractory diseases. However, studies have reported PTC resistance to chemotherapy. Therefore, the effect of chemotherapy on PTC was first determined in the present study.

**Figure 1:**
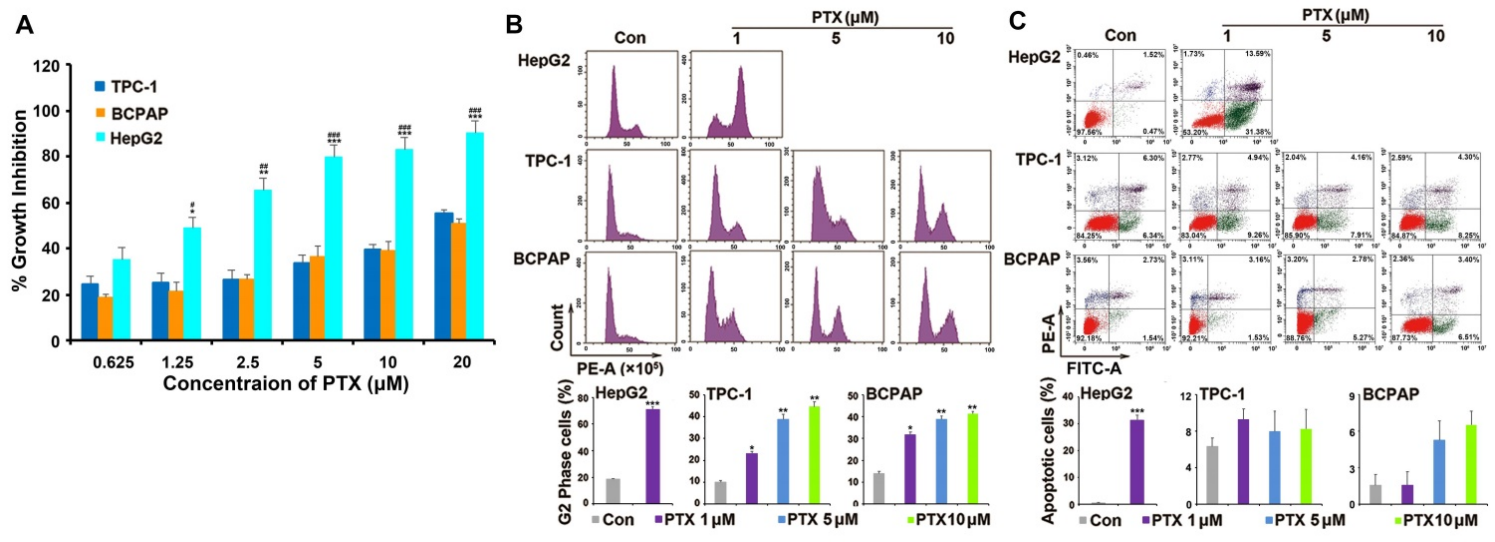
**PTC cells were resistant to PTX.** (**A**) XTT assay analyzed the effect of PTX on the proliferation in tumor cells. PTC cells (TPC-1 and BCPAP) and HepG2 cells were treated with different concentrations (0.625, 1.25, 2.5, 5, 10, and 20 μM) of PTX. An XTT assay was performed after 48 h to determine the relative proliferation rates and the IC_50_ values were calculated for PTX in the three cell types. *P<0.05, **P<0.01 and ***P<0.001, TPC-1 compared with HepG2; ^#^P<0.05, ^##^P<0.01 and ^###^P<0.001, BCPAP compared with HepG2. Flow cytometry analysis of (**B**) the cell cycle distributions and (**C**) apoptosis in TPC-1 and BCPAP cells treated with PTX. TPC-1 and BCPAP cells were treated with different concentrations of PTX (1, 5, and 10 μM), whereas HepG2 cells were treated with 1 μM PTX for 24 h and 48 h, respectively. Flow cytometry analysis of PI-stained cells was performed to determine the cell cycle distribution. Apoptosis was detected by flow cytometry staining with Annexin V-FITC (green) and PI (red). The percentage of apoptotic cells (Annexin V^+^/PI^-^) was determined using flow cytometry. ^*^P<0.05, ^**^P<0.01 and ^***^P<0.001, compared with control.

PTX is widely used as a broad-spectrum antitumor drug. Two PTC cell lines (TPC-1 and BCPAP) were chosen to investigate the sensitivity of PTC cells to PTX. One drug-sensitive tumor cell line (HepG2) was used as positive control cells. Compared with the response to PTX in HepG2 cells, TPC-1 and BCPAP cells were resistant to PTX. PTX inhibited the proliferation of HepG2 cells after treatment for 48 h, with a 50% inhibitory rate (IC_50_) of 1.25 μM. However, the concentration of PTX was 20 μM in TPC-1 and BCPAP cells (Fig. 1A).

### PTX did not induce apoptosis in PTC cells

Then, the underlying mechanism of drug resistance to PTX in PTC cells was investigated. As a microtubule polymerization enhancer, PTX performs antitumor activity by inducing G2/M phase accumulation and apoptosis in tumor cells 21. Because HepG2 cells were much more sensitive than PTC cells to PTX, we found that 1 μM PTX induced significant G2/M phase accumulation after treatment for 24 h and apoptosis following treatment for 48 h in HepG2 cells. However, different concentrations (1, 5 and 10 μM) of PTX induced G2/M phase accumulation after 24 h of treatment (Fig. 1B), they could not induce apoptosis after treatment for 48 h in TPC-1 and BCPAP cells (Fig. 1C). Moreover, 1, 5 and 10 μM PTX induced G2/M phase accumulation at 48 h in the two PTC cells (Fig. S1). This suggested that the mechanism of PTC cells resistance to PTX was associated with the inability of PTX to induce apoptosis of PTC cells.

### rAd-p53 increased the sensitivity of PTC cells to PTX

**Figure 2:**
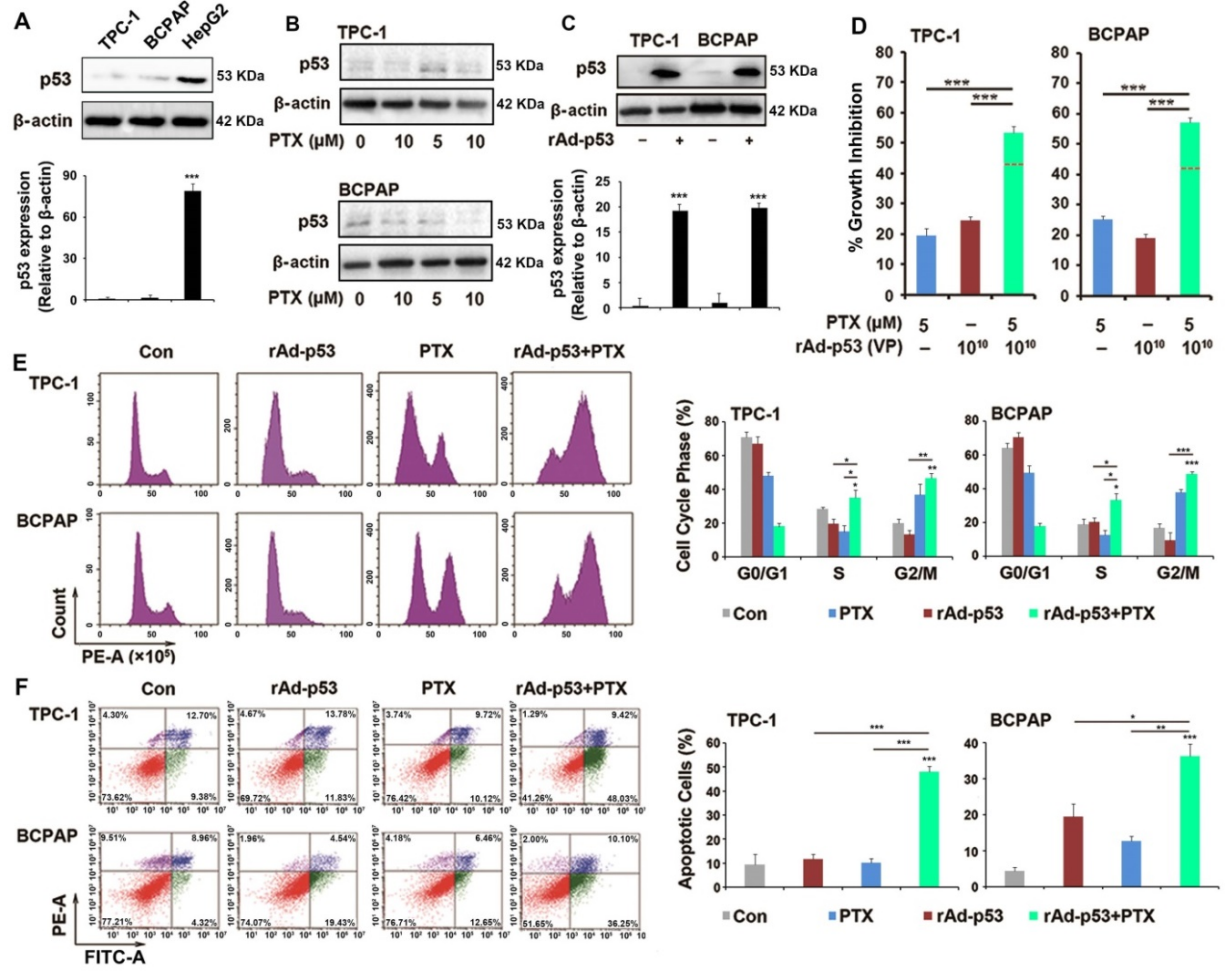
**rAd-p53 increased the sensitivity of PTC cells to PTX.** (**A-C**) Western blotting was performed to analyze the expression of p53 protein in PTC cells. ***P<0.001. (**D**) rAd-p53 enhanced the antitumor activity of PTX in TPC-1 and BCPAP cells. TPC-1 and BCPAP cells were treated with rAd-p53, PTX and rAd-p53 + PTX for 48 h. The dotted line denotes the predicted additivity [(eA + eB) - (eA x eB)] (eA, the inhibitory rate of A; eB, the inhibitory rate of B). ***P<0.001, compared with control. Flow cytometry was performed to determine (**E**) cell cycle distributions (cells stained with PI) and (**F**) the percentage of apoptotic cells (cells stained with Annexin V/PI) in TPC-1 and BCPAP cells. Cells were treated with vehicle, 10^10^ VP rAd-p53, 5 μM PTX or 10^10^ VP rAd-p53 + 5 μM PTX for 24 h or 48 h. ^*^P<0.05, ^**^P<0.01 and ^***^P<0.001, compared with control.

Previous studies have reported that the inactivation of p53 plays an important role in drug resistance and apoptosis in various tumors types 19,22. When the status of the *P53* gene was previously determined in TPC-1 and BCPAP cells, it was found that wt-p53 could be detected in TPC-1 cells and p53 mutant could be detected in BCPAP cells 23. However, the western blotting results of the present study showed that p53 expression was low in TPC-1 and BCPAP cells (Fig. 2A) and PTX could not increase the expression of p53 in the two PTC cells (Fig. 2B). rAd-p53 was used to increase the expression of wt-p53 in tumor cells (Fig. 2C). Because 0.1% DMSO and adenovirus vector both had no interfering effect in PTC cells in preliminary experiment (Fig. S2), just adenovirus vector-treated group was used as control group in next studies. We had done the preliminary experiments to make sure that 10^10^ VP was the most appropriate concentration of rAd-p53 (Table S1). 10^10^ VP rAd-p53 markedly increased the sensitivity of PTC cells to PTX (Fig. 2D). 10^10^ VP rAd-p53 + 5 μM PTX led to >50% inhibition of cell proliferation (54.02 ± 0.43% and 57.72 ± 0.38%) of TPC-1 and BCPAP cells, while 5 μM PTX treatment alone caused ~20% inhibition of cell proliferation in the two PTC cell lines (Fig. 2D). 10^10^ VP rAd-p53 combined with 5 μM PTX or 10 μM PTX showed sensitization in TPC-1 and BCPAP cells (the predicted additivity is shown with the dotted line in Fig. 2D).

### Combined rAd-p53 and PTX treatment induced cell cycle arrest and apoptosis

Because 5 and 10 μM of PTX combined with 10^10^VP rAd-p53 revealed no significant difference (Table S2), we therefore chose the small dose (5 μM) of PTX to combine with rAd-p53 in current study. Cell cycle analysis showed that rAd-p53 + PTX not only increased the number of cells in G2/M phase accumulation, but also induced S phase accumulation in TPC-1and BCPAP cells (Fig. 2E). PTC cells treated with rAd-p53 + PTX had a higher percentage of S phase cells (TPC-1 cells, 35.22 ± 4.52% and BCPAP cells, 33.24 ± 4.12%) than those treated with rAd-p53 alone (19.67 ± 2.97% and 20.21 ± 2.61%; P<0.05) and PTX alone (14.99 ± 3.58% and 12.58 ± 3.12%; P<0.05) (Fig. 2E). Furthermore, it was observed that rAd-p53 + PTX induced obvious apoptosis in TPC-1 and BCPAP cells. The number of apoptotic cells was 48.03 ± 2.45% for TPC-1 and 36.25 ± 3.39% for BCPAP cells, which was higher than the control (9.38 ± 4.48% and 4.32 ± 1.09%; P < 0.001), rAd-p53-treated alone (11.83 ± 1.99% and 19.43 ± 3.62%; P<0.05), and PTX-treated (10.12 ± 1.78% and 12.66 ± 1.39%; P<0.01) groups (Fig. 2F).

### Combined rAd-p53 and PTX treatment induced S phase accumulation and apoptosis in a p53-dependent pathway response to DNA damage

**Figure 3:**
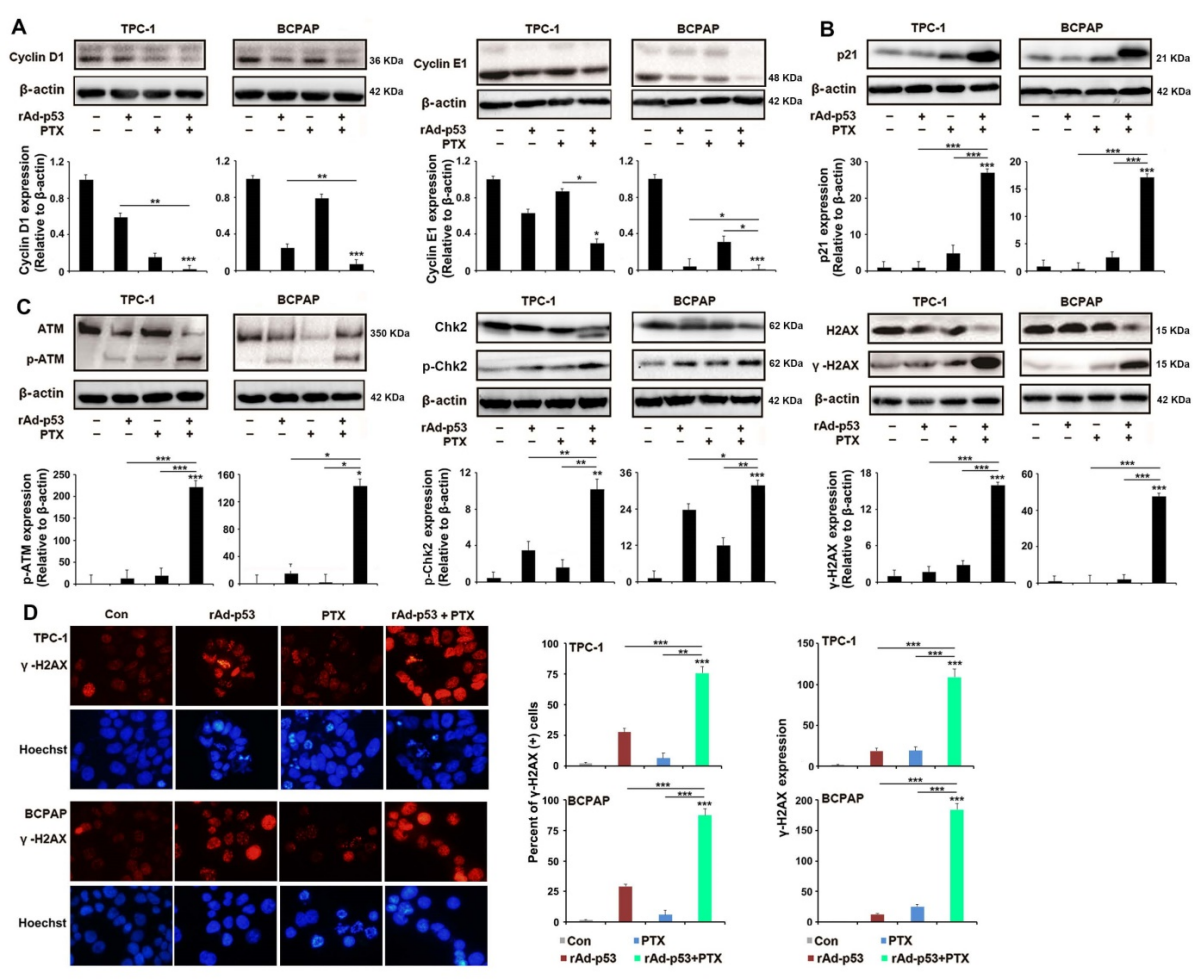
**rAd-p53 + PTX induced S arrest in a p53-dependent pathway in response to DNA damage in PTC cells.** (**A-C**) Representative western blotting images show the expression of (**A**) S phase cell cycle-related proteins, (**B**) p21 and (**C**) DNA damage-related proteins in TPC-1 and BCPAP cells. PTC cells were treated with vehicle, 10^10^ VP rAd-p53, 5 μM PTX, or 10^10^ VP rAd-p53 + 5 μM PTX for 24 h. *P<0.05; **P<0.01 and ***P<0.001, compared with control. (**D**) Representative immunofluorescence images (magnification, x200) show γ-H2AX-stained TPC-1 and BCPAP cells. PTC cells were treated with vehicle, 10^10^ VP rAd-p53, 5 μM PTX, or 10^10^ VP rAd-p53 + 5 μM PTX for 24 h. Nuclei were stained with Hoechst 33258.

Then, the signaling pathways that induce the S phase cell cycle arrest and apoptosis-induced by rAd-p53 + PTX in PTC cells were analyzed. Cyclins and Cyclin-dependent kinase (CDK) are two main types of proteins which involved in regulation of cell cycle progression. CDK4 and -6, which along with their Cyclins (Cyclin D1 and Cyclin E1 belong to the G1 cyclins), regulate the passage of cells through the G1 phase and their entry into the S phase 24,25. Western blotting showed that rAd-p53 + PTX decreased the expression levels of cyclin D1 and -E1 protein (Fig. 3A), but had no effect on the expression of CDK 4 and -6 proteins (Fig. S3) in the PTC cells. As a main transcriptional regulator, p53 could modulates the cell cycle progression through regulating p21 expression. p21 inhibits cyclin E1-CDK2 and cyclin D1-CDK4,6 complexes and induces cells G1 and S phases accumulation 26-28. p21 protein expression was notably increased in the rAd-p53 + PTX-treated TPC-1 and BCPAP cells (Fig. 3B). These results demonstrated that rAd-p53 + PTX treatment induced S phase accumulation in PTC cells by activating the p53/p21 pathway, which resulted in decreased expression levels of cyclin D1 and E1.

In most mammalian cells, cell cycle arrest is always induced by DNA damage in response to cellular stress 25. Therefore, the status of the DNA damage response in PTC cells was analyzed in the present study. Western blotting analysis showed that rAd-p53 + PTX increased the expression levels of phosphorylated-ATM, phosphorylated-Chk2 and γ-H2AX proteins in PTC cells (Fig. 3C). Immunofluorescence analysis confirmed the highest level of γ-H2AX in the rAd-p53 + PTX-treated PTC cells (Fig. 3D). This suggested that rAd-p53 + PTX activated the DNA damage signaling pathway in PTC cells.

**Figure 4:**
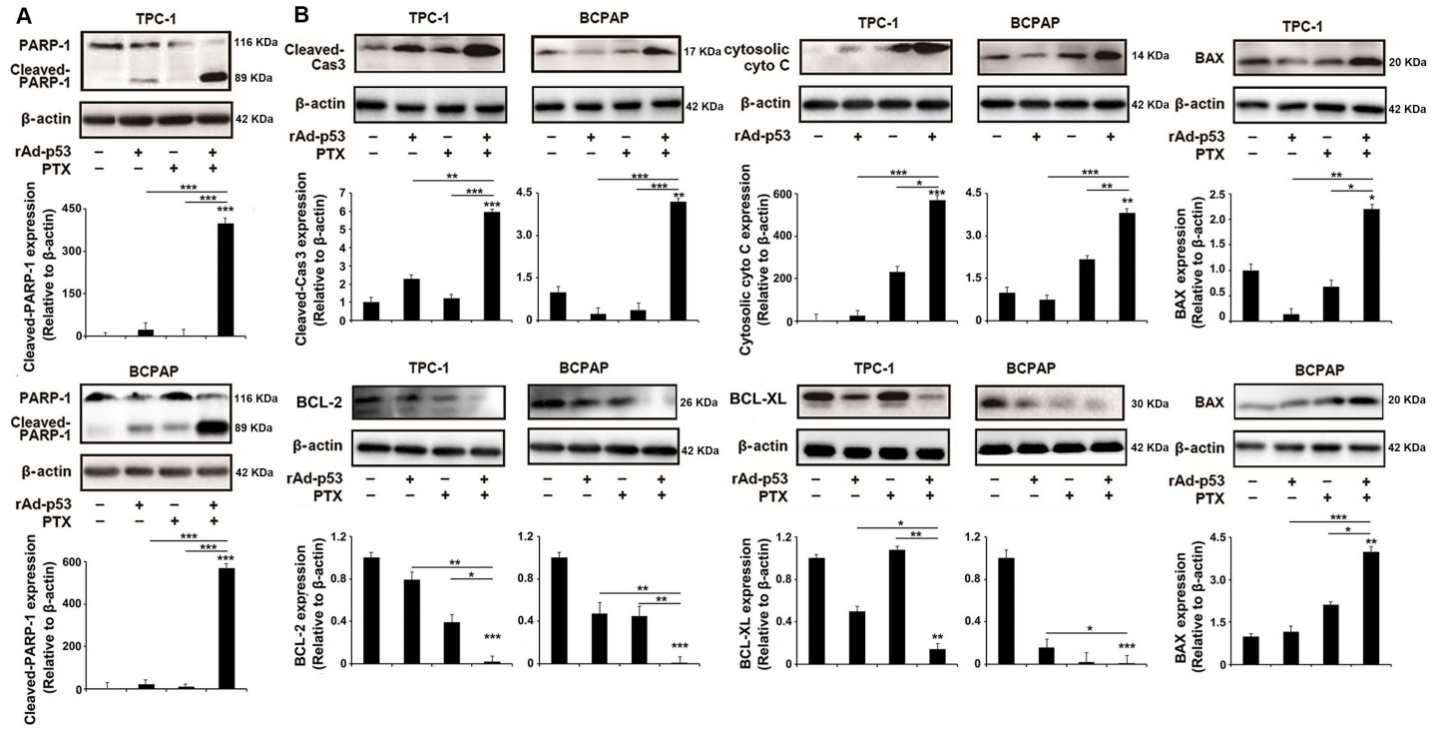
**rAd-p53 + PTX induced DNA damage-induced apoptosis in PTC cells.** Western blotting was performed to analyze the levels of (**A**) cleaved PARP-1 and (**B**) apoptotic pathway-related proteins in TPC-1 and BCPAP cells. PTC cells were treated with vehicle, 10^10^ VP rAd-p53, 5 μM PTX, or 10^10^ VP rAd-p53 + 5 μM PTX for 48 h. ^*^P<0.05, ^**^P<0.01 and ^***^P<0.001, compared with control.

When DNA damage is irreparable, mammalian cells undergo programmed cell death *via* the apoptotic pathway 29. PARP-1, as a critical DNA damage repair protein, is cleaved by cleaved Caspase 3-during DNA damage-induced apoptosis 11,30. As shown in Fig. 4A, the level of cleaved PARP-1 was notably increased in rAd-p53 + PTX-treated PTC cells, suggesting that rAd-p53 + PTX promoted DNA damage-induced apoptosis in PTC cells. Western blotting analysis showed that rAd-p53 + PTX did increase the level of cleaved Caspase 3 in PTC cells (Fig. 4B). As one of the critical determinants of cell survival in response to DNA damage, p53 activates the expression of downstream genes such as BCL-2 family proteins to activate mitochondrial apoptotic pathway 14,31. BCL-2 and BCL-XL are apoptotic inhibitors that decreases the accumulation of cytosolic cytochrome C, which activates caspase 3 in DNA damage-induced apoptosis 12. In the present study the expression of these apoptosis-related proteins was measured in PTC cells. The results showed that rAd-p53 + PTX increased the levels of cytosolic cytochrome C and BAX proteins and decreased the levels of BCL-2 and BCL-XL proteins in PTC cells (Fig. 4B).

### Combined rAd-p53 and PTX treatment inhibited tumor growth of TPC-1 xenograft tumors in nude mice

**Figure 5:**
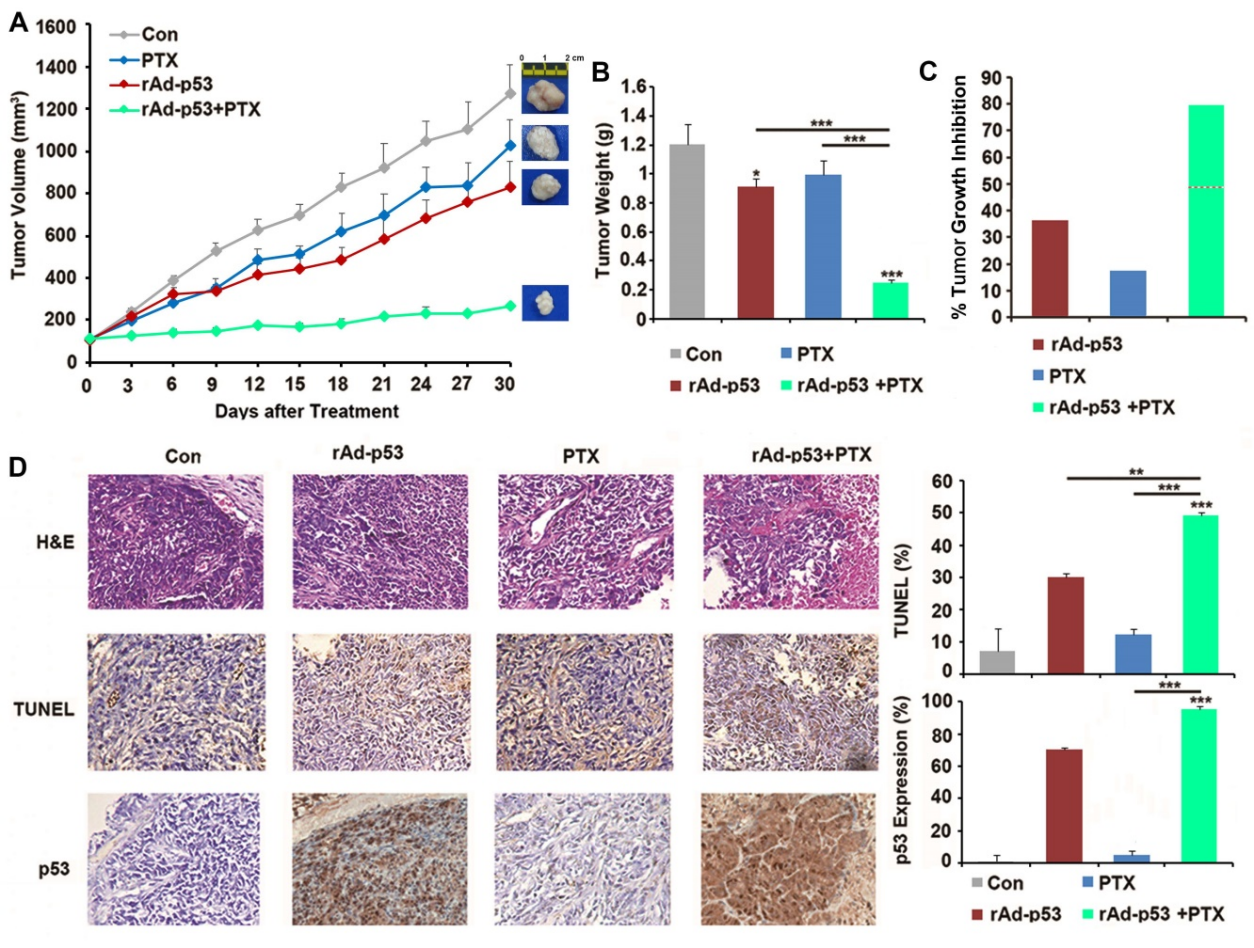
**Wt-p53 overexpression enhanced antitumor activity of PTX in the TPC-1 xenograft mouse model.** (**A**) TPC-1 xenograft tumor volume curves for mice in the control, rAd-p53, PTX, and rAd-p53 + PTX groups (n = 6/group). (**B**) Bar chart showing the tumor weights in the control, rAd-p53, PTX, and rAd-p53 + PTX groups (n = 6/group). **P<0.01 and ***P<0.001. (**C**) Bar chart showing the inhibitory rate of tumor development in the rAd-p53, PTX and rAd-p53 + PTX groups mice relative to the control group. The dotted line denotes predicted additivity [(eA + eB) - (eA x eB)]. (**D**) Representative images (magnification, x100) of hematoxylin & eosin (H&E)- and TUNEL-stained tumor sections showing tumor necrosis and apoptosis. Immunohistochemical staining (magnification, x100) of p53 proteins in tumor sections belonging to the four groups. ^**^P<0.01 and ^***^P<0.001, compared with control.

A xenograft mouse model was generated to further investigate the inhibitory effect of rAd-p53 + PTX on tumor growth in PTC. The results showed that rAd-p53 + PTX significantly inhibited tumor growth in a TPC-1 xenograft model. The tumor volume in the rAd-p53 + PTX treatment group was the lowest (265.91 ± 9.24 mm^3^) of all treatment groups [compared with vehicle control group (1257.27 ± 134.29 mm^3^), P<0.001; compared with rAd-p53-treated group (779.16 ± 123.51 mm^3^), P = 0.005; compared with PTX-treated group (1026.63 ± 122.29 mm^3^), P<0.001] (Fig. 5A). The tumor inhibitory rates of the rAd-p53, PTX, and rAd-p53 + PTX treatment groups relative to the vehicle control group were 40.30, 17.45, and 79.39%, respectively (Fig. 5B and C).

The results of the TUNEL assay demonstrated that rAd-p53 + PTX treatment induced apoptosis in the xenograft tumors (Fig. 5D). Immunohistochemical analysis revealed that there was no expression of p53 protein in the control group, low expression in PTX-treated tumors, and high expression in rAd-p53-treated and rAd-p53 + PTX-treated tumors (Fig. 5D).

The results of *in vivo* experiment indicated that PTX treatment alone could not inhibit tumor growth, but rAd-p53 + PTX significantly inhibited tumor growth in PTC, which indicated that p53 activation was necessary for the antitumor activity of PTX in PTC.

## Discussion

Refractory types of PTC, including radioiodine refractory diseases, have poor prognosis rates due to the limitation of traditional treatment approaches 2,3. Chemotherapy could be a potential strategy for these cases. However, various questions still need answering, for example what is the nature of PTC tumors? Are there any drug-sensitive pathways in PTC tumors? Thus, studying the pathology and pharmacology of PTC tumors is of great significance.

Apoptotic pathways are important for cellular activities and functions in tumor cells, and resistance to apoptosis may be a contributing factor in the development of chemoresistance in tumor cells 32. A previous study reported that chemotherapeutic drugs that target apoptotic pathways would make tumor cells sensitive to chemotherapy and radiotherapy 33. In the present study, the flow cytometry results showed that PTX only induced G2/M phase arrest, but not apoptosis in PTC cells. Under this condition, 10 μM PTX could not inhibit proliferation in 50% of PTC cells. However, treatment with rAd-p53 + 5 μM PTX induced obvious apoptosis in PTC cells, while they notably inhibited cells proliferation, with an inhibitory rate of >50%. rAd-p53 also significantly increased the antitumor activity of PTX* in vivo* by inducing tumor apoptosis. The results confirmed that resistance to apoptosis mediated PTC cells resistance to PTX.

Next, which signaling pathway or regulators were inactivated in the PTX-induced apoptotic pathway in PTC cells? Studies have reported that inactivation of p53 might contribute to resistance to cancer drugs and inhibit apoptosis of tumor cells 19. In the present study, the status of the *P53* gene was found to be the wt-p53 in TPC-1 cells and p53 mutant in BCPAP cells. Compared with in p53-inactivated PTC cells whereby PTX induced G2/M arrest by targeting the microtubule polymerization, PTX induced obvious S arrest and apoptosis in p53-activated PTC cells (Fig. 2E,F), suggesting that activation of p53 played an important role in the mechanism of antitumor action of PTX in PTC, and PTX induced S phase arrest and apoptosis in a p53-dependent pathway in PTC cells. In PTC cells, treatment with rAd-p53 increased the expression of wt-p53 protein. rAd-p53 + PTX activated p21 to negatively regulate Cyclin D1/E1 to induce S phase arrest in response to DNA damage within 24 h and regulated the expression of BAX, BCL-2 and BCL-XL to mediate the DNA damage-induced apoptosis after 48 h of treatment.

In summary, the results in the present study showed that p53-dependent pathway was necessary for the inhibition of PTX in PTC cells. The result that rAd-p53 + PTX significantly inhibited PTC tumor *in vitro* and *in vivo* through p53-dependent apoptosis response to DNA damage suggested activation of p53 had a great meaning for the clinical management and treatment of PTC and rAd-p53 combined with chemotherapy, which might be used in the treatment of advanced-stage PTC patients with tumors unsuitable for surgery.

## Supplementary Material

Supplementary figures and tables.
