# Supplementary material for: p53-dependent apoptosis is essential for the antitumor effect of paclitaxel response to DNA damage in papillary thyroid carcinoma
Source: Int J Med Sci. 2021 Jul 11;18(14):3197–205. doi: 10.7150/ijms.61944 (PMC8364467; doi:10.7150/ijms.61944)
Supplement: Supplementary file 1 — Supplementary figures and tables. [file ijmsv18p3197s1.pdf]

Figure S1

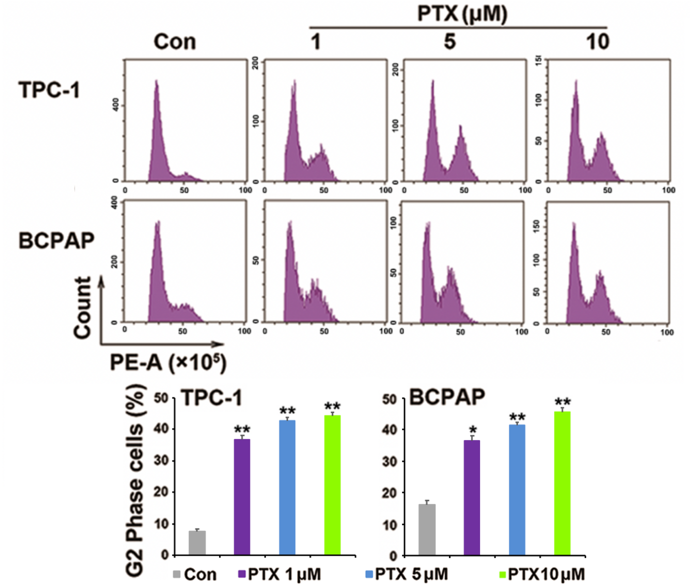

Figure S1. PTX induces G2/M phase cell cycle arrest at 48 h in TPC-1 and BCPAP cells. TPC-1 and BCPAP cells were treated with different concentrations of PTX (1, 5, and 10 μM) for 48 h. Control cells were treated with 0.1% DMSO. Flow cytometry analysis of PI-stained cells was performed to determine the cell cycle distribution. $^{*}P<0.05$, $^{**}P<0.01$. PTX, paclitaxel; PI, propidium iodide.

Figure S2

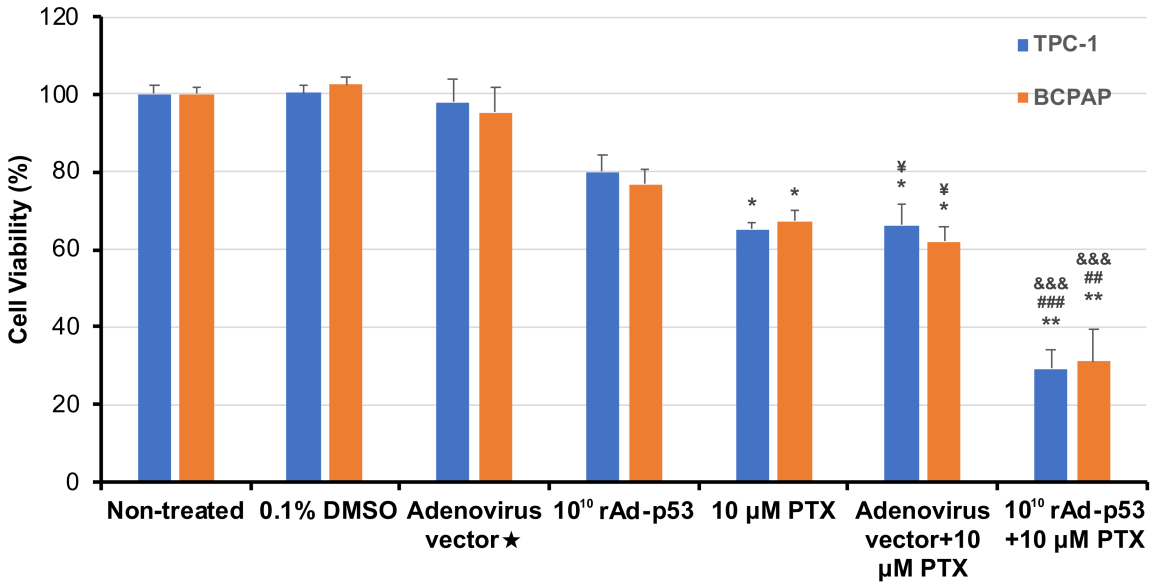

Figure S2. The inhibitory rate of different treatments in PTC cells. ★: The volume of Adenovirus vector which was added into medium was as some as 10^{10}VP rAd-p53. $^{*}P<0.05$ and $^{**}P<0.01$: compared with non-treated group; $^{¥}P<0.05$: compared with Adenovirus vector-treated group; $^{\#\#}P<0.01$ and $^{\#\#\#}P<0.001$: compared with rAd-p53-treated group; $^{\&\&\&}P<0.001$: compared with 10 μM PTX-treated group. DMSO, dimethyl sulfoxide; PTX, paclitaxel; rAd-p53, recombinant adenovirus-p53.

Figure S3

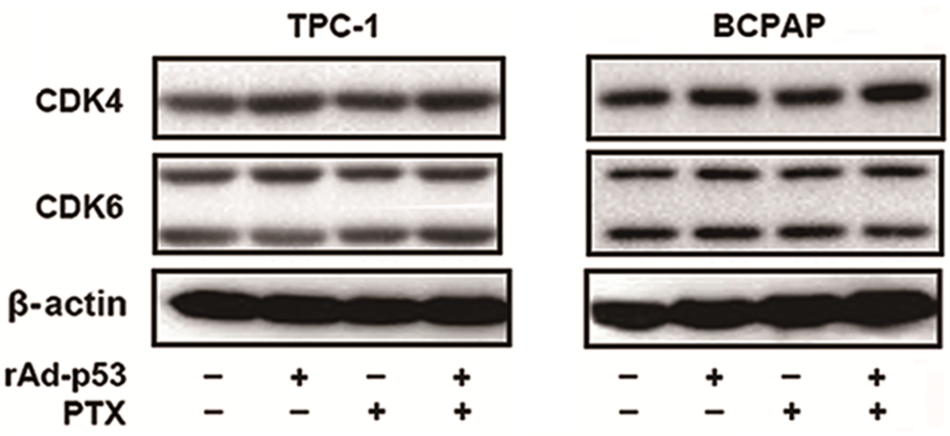

Figure S3. rAd-p53 combined with PTX has no effect on the expression levels of CDK4 and CDK6 proteins in TPC-1 and BCPAP cells. PTX, paclitaxel; rAd-p53, recombinant adenovirus-p53.

Table S1

Table S1 The inhibitory rate of different concentrations of rAd-p53 combined with/without 10 μM PTX in PTC cells.

Cell Line			TPC-1	BCPAP
Inhibitory Rate (%)	Concentrations of rAd-p53	10^9	4.97 ± 1.85	12.84 ± 2.39
		10^{10}	22.76 ± 5.42	19.48 ± 3.28
	10 μM PTX combined with different concentrations of rAd-p53	0	34.36 ± 3.45	31.98 ± 6.97
		10^9	38.93 ± 4.98	36.05 ± 3.31
		10^{10}	67.93 ± 7.06**	65.51 ± 5.95***

**P < 0.01 and ***P < 0.001; 10^{10}VP rAd-p53 combined with 10 μM PTX compared with 10^{10}VP rAd-p53. PTX, paclitaxel; rAd-p53, recombinant adenovirus-p53.

Table S2

Table S2 The inhibitory rate of different concentrations of PTX combined with/without 10^{10}VPrAd-p53 in PTC cells.

**Cell Line**			**TPC-1**	**BCPAP**
Inhibitory Rate (%)	Concentrations of PTX (μM)	1	17.95 ± 2.41	16.96 ± 3.29
		5	34.38 ± 8.72	34.09 ± 3.83
		10	38.04 ± 9.35	33.98 ± 8.43
	10^{10}VP rAd-p53combined with different concentrations of PTX	0	26.05 ± 2.71	15.48 ± 2.59
		1	20.90 ± 3.98	26.37 ± 0.91
		5	$59.09 \pm 9.83^{**}$	$61.13 \pm 8.97^{*}$
		10	$62.87 \pm 7.77^{\#}$	$67.75 \pm 9.39^{\#\#}$

*P < 0.05 and **P < 0.01; 10^{10}VP rAd-p53 combined with 5 μM PTX compared with 5 μM PTX; $^{\#}$P < 0.05 and $^{\#\#}$P < 0.01; 10^{10}VP rAd-p53 combined with 10 μM PTX compared with 10 μM PTX. PTX, paclitaxel; rAd-p53, recombinant adenovirus-p53.
